# Non-cancer to anti-cancer: investigation of human ether-a-go-go-related gene potassium channel inhibitors as potential therapeutics

**DOI:** 10.1186/s43046-021-00091-3

**Published:** 2021-11-08

**Authors:** Vaishali M. Patil, Anand Gaurav, Priyanka Garg, Neeraj Masand

**Affiliations:** 1 KIET School of Pharmacy, KIET Group of Institutions, Delhi-NCR, Ghaziabad, India; 2 Faculty of Pharmaceutical Sciences, UCSI University, Jalan Menara Gading, Taman Connaught, Cheras, 56000 Kuala Lumpur, Malaysia; 3 Department of Pharmacy, Lala Lajpat Rai Memorial Medical College, Meerut, Uttar Pradesh India

**Keywords:** hERG inhibitors, Non-cancer, Anti-cancer agents, Molecular docking, Drug repurposing

## Abstract

**Background:**

The expression of hERG K^+^ channels is observed in various cancer cells including epithelial, neuronal, leukemic, and connective tissue. The role of hERG potassium channels in regulating the growth and death of cancer cells include cell proliferation, survival, secretion of proangiogenic factors, invasiveness, and metastasis.

**Methods:**

In the reported study, an attempt has been made to investigate some non-cancer hERG blockers as potential cancer therapeutics using a computational drug repurposing strategy. Preliminary investigation for hERG blockers/non-blockers has identified 26 potential clinically approved compounds for further studies using molecular modeling.

**Results:**

The interactions at the binding pockets have been investigated along with the prioritization based on the binding score. Some of the identified potential hERG inhibitors, i.e., Bromocriptine, Darglitazone, and Troglitazone, have been investigated to derive the mechanism of cancer inhibition.

**Conclusions:**

The proposed mechanism for anti-cancer properties via hERG blocking for some of the potential compounds is required to be explored using other experimental methodologies. The drug repurposing approach applied to investigate anti-cancer therapeutics may direct to provide a therapeutic solution to late-stage cancer and benefit a significant population of patients.

## Background

The human ether-a-go-go-related gene potassium channel (hERG, Kv11.1, KCNH2) are voltage-dependent K+ channels. These are expressed in cardiac myocytes, neurons, smooth muscles, and neuroendocrine cells [1]. In cardiac cells, hERG contributes to action potential repolarization [2] and their dysfunction is associated with lethal ventricular arrhythmias. While in case of anti-cancer drug discovery, some ion channels are involved in signaling pathways which lead to cell proliferation or apoptosis and are considered as major targets of interest [3–6].

The expression of hERG K^+^ channels is observed in various cancer cells (epithelial, neuronal, leukemic, and connective tissue). When compared to corresponding non-cancerous cells, the hERG protein levels were found to be absent. The aberrant expression of hERG in neoplastic cells and primary human cancers (glioma, neural crest-derived tumors, carcinomas, and leukemias) has been reported [7]. The role of hERG potassium channels in regulating the growth and death of cancer cells has been investigated. It includes cell proliferation, survival, secretion of proangiogenic factors, invasiveness, and metastasis [7, 8]. Some of the important reviews focusing on the expression, function, and regulation in proliferation and apoptosis have been published [7–10]. Studies conclude three major functions of hERG related to tumor cell biology which include regulation of cell proliferation (in leukemia), the control of tumor cell invasiveness through physical and functional interactions with adhesion receptors, and regulating tumor cell neoangiogenesis by modulating the angiogenic factor secretion [8, 11]. For the diagnosis and treatment of human cancers, hERG channels are considered as the novel targets. In the last decade, drug repurposing strategies have been successfully implemented including evaluation of non-cancer therapeutics as hERG blockers and later as potential anti-cancer agents. Studies have been carried out for evaluating the hERG K^+^ channel inhibitors having cell cycle arrest properties namely, E-4031, WAY 123398, CsCl, HERG-specific siRNA, Doxazosin (antihypertensive α_1_-adrenoreceptor blocker), Astemizole, Erythromycin (a macrolide antibiotic), and Terazosin [8]. Successful application of hERG inhibitors as anti-cancer therapeutics requires careful evaluation due to the associated risk of proarrhythmia and cardiotoxicity [2]. Thus, the use of hERG inhibitors as anti-cancer agents may cause apoptosis, heart failure, QT prolongation, and ventricular tachycardia. Looking towards the application of anti-cancer therapeutics in life-threatening situations, the potential cardiac toxicity is acceptable with a short-term use of hERG inhibitors in the treatment of cancer.

**Fig. 1:**
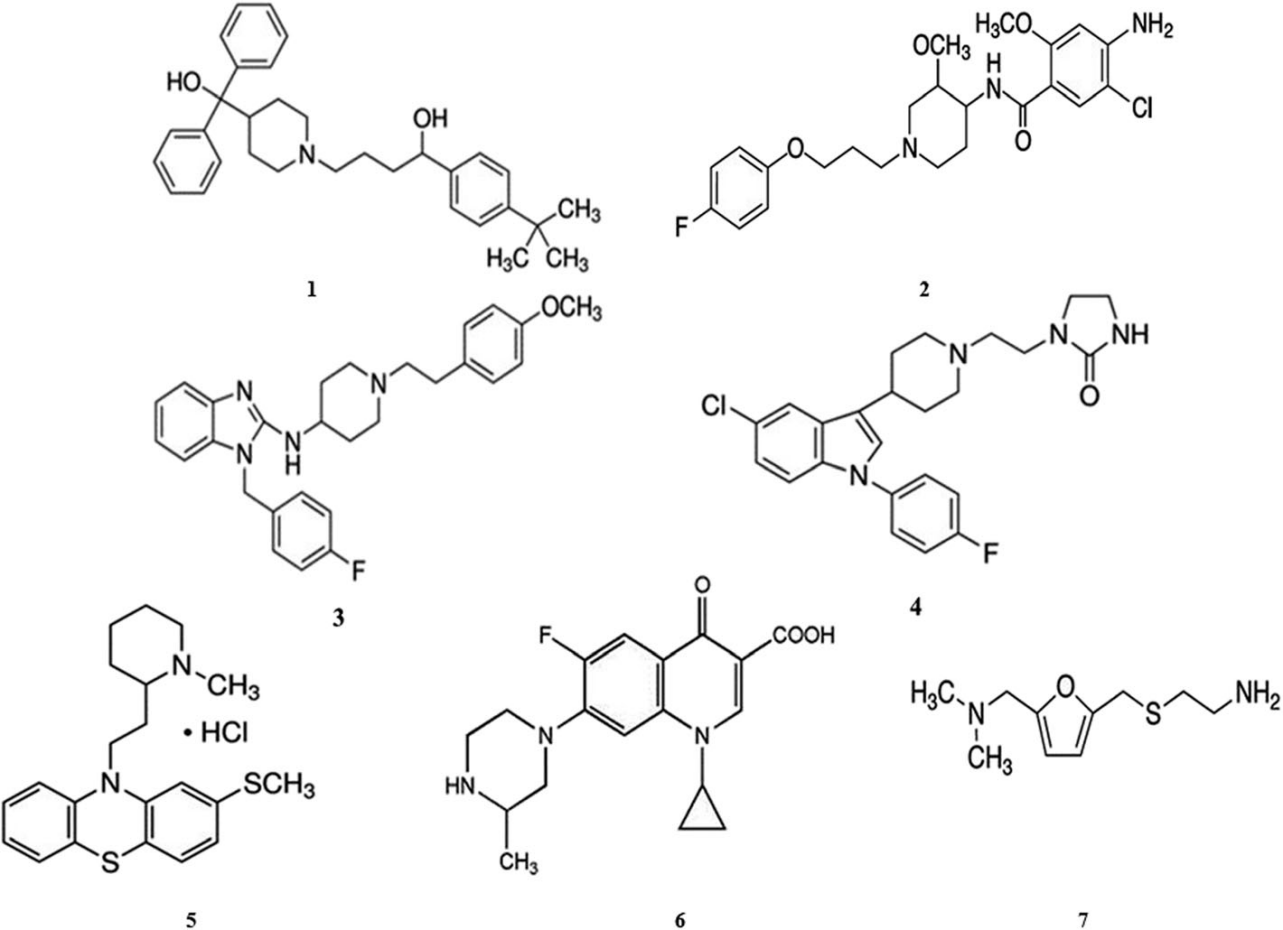
Chemical structures of Terfenadine (**1**), Cisapride (**2**), Astemizole (**3**), Sertindole (**4**), Thioridazine (**5**), Grepafloxacin (**6**), and Ranitidine (**7**)

The drugs are being routinely evaluated for potential cardiotoxic effects to avoid clinical failures as part of the drug discovery process. Some of the examples in this series are terfenadine, cisapride, astemizole, sertindole, thioridazine, grepafloxacin, and ranitidine (Fig. 1) [12]. The early-stage prediction of potential toxicity can be easily evaluated using the prediction models such as pred-hERG [13]. The cardiac toxicity prediction model is based on the mechanism of drug binding to the cardiac potassium channel encoded by the human *ether-a-go-go*-related gene (hERG). Inhibition of hERG causes long QT syndrome (LQTS) leading to fatal ventricular arrhythmias and sudden death [2, 14].

hERG inhibition retards cardiac repolarization causing arrhythmia, ventricular fibrillation. Computational tools have been reported to be used for predicting hERG-related cardiotoxicity of drug candidates [15, 16]. Some drugs from class III anti-arrhythmic agents cause arrhythmia by hERG inhibition. Examples of such drugs are E4031, Way 123, 398 and dofetilide. In recent years, antidiabetic drugs have been investigated for their pharmacotherapy and incidences of cancer [17]. Some of them have shown promising results during anti-cancer evaluations and its examples include metformin (cytostatic effect) [18], Glitazones (promote apoptosis), α-glucosidase inhibitors, and SGLT2 inhibitors [17]. Subsequent studies have demonstrated a higher risk of cancer (liver, pancreas, endometrium, colon, breast, bladder, and colorectal) among diabetic patients [19].

With regard to understanding the anticancer mechanism of antidiabetic drugs acting through blockage of hERG channel, the present work based on the use of computational methods is planned. In the present manuscript, a dataset of anti-diabetic drugs has been evaluated for hERG inhibition properties and identified inhibitors were studied for binding interactions at the receptor sites. In view of the role of hERG in cancer, the preliminary work reported in this manuscript has been planned. The study aims to apply a drug repurposing approach to identify potential anti-cancer lead/drug candidates from a small dataset of non-cancer drugs.

## Methods

### Data set

Drugs from several classes have been approved for clinical use in the treatment of diabetes. Some of the major categories which have been approved by the United States Food and Drug Administration (US FDA) include biguanides, sulfonylureas, meglitinides, thiazolidinediones, dipeptidyl peptidase IV (DPP IV) inhibitors, glucagon-like peptide-1 (GLP-1), and α-glucosidase inhibitors. Among them, 77 compounds which have been approved for clinical use were used to create a small dataset (Table 1) [20–22]. Different search engines namely, PubMed, MEDLINE, EMBASE, and Google Scholar, were used to collect the dataset including chemical structures.

**Table 1:** Details of anti-diabetic compounds selected for study and their hERG blocking profile

Drug	Prediction/potency	Confidence	Applicability domain (AD)
Acarbose	Potential cardiotoxic (+)	50%	No (Value =0.16, limit=0.26)
	Weak/moderate	50%	
Alrestatin	Non-cardiotoxic (**−**)	60%	No (Value =0.24, limit=0.26)
	Not applicable		
Acetohexamide	Non-cardiotoxic (**−**)	80%	Yes (Value =0.31, limit=0.26)
	Not applicable		
Anagliptin	Non-cardiotoxic (**−**)	80%	Yes (Value =0.29, limit=0.26)
	Not applicable		
Alogliptin	Non-cardiotoxic (**−**)	60%	Yes (Value =0.39, limit=0.26)
	Not applicable		
Buformin	Non-cardiotoxic (**−**)	**50**	No (Value =0.16, limit=0.26)
	Not applicable		
Bromocriptine	**Potential cardiotoxic (+)**	**60**	Yes (Value =0.26, limit=0.26)
	**Weak/moderate**	**50**	
Balaglitazone	**Potential cardiotoxic (+)**	**60**	Yes (Value =0.34, limit=0.26)
	**Weak/moderate**	**60**	
Carbutamide	Non-cardiotoxic (**−**)	**70**	Yes (Value =0.29, limit=0.26)
	Not applicable		
Canagliflozin	Non-cardiotoxic (**−**)	**50**	No (Value =0.25, limit=0.26)
	Not applicable		
Ciglitazone	Non-cardiotoxic (**−**)	**50**	Yes (Value =0.26, limit=0.26)
	Not applicable		
Chlorpropamide	Non-cardiotoxic (**−**)	**70**	Yes (Value=0.31, limit=0.26)
	Not applicable		
Darglitazone	**Potential cardiotoxic (+)**	**50**	Yes (Value =0.3, limit=0.26)
	**Weak/moderate**	**60**	
Empagliflozin	**Potential cardiotoxic (+)**	**60**	Yes (Value =0.28, limit=0.26)
	**Strong/extreme**	**50**	
Dapagliflozin	**Potential cardiotoxic (+)**	**60**	Yes (Value =0.29, limit=0.26)
	**Strong/extreme**	**50**	
Englitazone	**Potential cardiotoxic (+)**	**60**	Yes (Value =0.28, limit=0.26)
	**Strong/extreme**	**50**	
Epalrestat	Non-cardiotoxic (**−**)	**60**	No (Value =0.24, limit=0.26)
	Not applicable		
Ertugliflozin	Non-cardiotoxic (**−**)	**60**	Yes (Value =0.3, limit=0.26)
	Not applicable		
Euogliptin	Non-cardiotoxic (**−**)	**80**	Yes (Value =0.28, limit=0.26)
	Not applicable		
Fidarestat	Non-cardiotoxic (**−**)	**60**	No (Value =0.25, limit=0.26)
	Not applicable		
Glibenclamide	Non-cardiotoxic (**−**)	**80**	Yes (Value =0.34, limit=0.26)
	Not applicable		
Gemigliptin	Non-cardiotoxic (**−**)	**60**	No (Value =0.24, limit=0.26)
	Not applicable		
Gliclazide	Non-cardiotoxic (**−**)	**70**	Yes (Value =0.3, limit=0.26)
	Not applicable		
Glibornuride	Non-cardiotoxic (**−**)	**70**	Yes (Value =0.28, limit=0.26)
	Not applicable		
Glimepride	Non-cardiotoxic (**−**)	**70**	No (Value =0.31, limit=0.26)
	Not applicable		
Gliquidone	Non-cardiotoxic (**−**)	**50**	Yes (Value =0.35, limit=0.26)
	Not applicable		
Glipizide	Non-cardiotoxic (**−**)	**60**	Yes (Value =0.32, limit=0.26)
	Not applicable		
Gosogliptin	Non-cardiotoxic (**−**)	**90**	Yes (Value =0.29, limit=0.26)
	Not applicable		
Glyclopyramide	Non-cardiotoxic (**−**)	**70**	Yes (Value =0.35, limit=0.26)
	Not applicable		
Glisoxepide	Non-cardiotoxic (**−**)	**50**	Yes (Value =0.33, limit=0.26)
	Not applicable		
Imirestat	Non-cardiotoxic (**−**)	**60%**	No (Value =0.24, limit=0.26)
	Not applicable		
Linogliride	Non-cardiotoxic (**−**)	**70%**	Yes (Value =0.27, limit=0.26)
	Not applicable		
Linagliptin	Non-cardiotoxic (**−**)	**80%**	Yes (Value =0.4, limit=0.26)
	Not applicable		
Lidorestat	Non-cardiotoxic (**−**)	**60%**	No (Value =0.25, limit=0.26)
	Not applicable		
Ipragliflozin	**Potential cardiotoxic (+)**	**50%**	No (Value =0.23, limit=0.26)
	**Weak/Moderate**	**50%**	
Lobeglitazone	**Potential cardiotoxic (+)**	**60%**	Yes (Value =0.27, limit=0.26)
	**Weak/Moderate**	**60%**	
Luseogliflozin	**Potential cardiotoxic (+)**	**50%**	Yes (Value =0.28, limit=0.26)
	**Weak/moderate**	**50%**	
Meglitinide	**Potential cardiotoxic (+)**	**50%**	Yes (Value =0.32, limit=0.26)
	**Weak/moderate**	**60%**	
Metahexamide	Non-cardiotoxic (**−**)	**80%**	Yes (Value =0.3, limit=0.26)
	Not applicable		
Metformin	Non-cardiotoxic (**−**)	**70%**	No (Value =0.14, limit=0.26)
	Not applicable		
Mifepristone	**Potential cardiotoxic (+)**	**60%**	No (Value =0.21, limit=0.26)
	**Weak/moderate**	**50%**	
Miglitol	Non-cardiotoxic (**−**)	**70%**	No (Value =0.16, limit=0.26)
	Not applicable		
Mitiglinide	Non-cardiotoxic (**−**)	**60%**	Yes (Value =0.29, limit=0.26)
	Not applicable		
Minalirestat	**Potential cardiotoxic (+)**	**50%**	Yes (Value =0.26, limit=0.26)
	**Weak/moderate**	**60**	
Nateglinide	Non-cardiotoxic (**−**)	**60%**	Yes (Value =0.31, limit=0.26)
	Not applicable		
Netoglitazone	Non-cardiotoxic (**−**)	**50%**	Yes (Value =0.28, limit=0.26)
	Not applicable		
Omarigliptin	Non-cardiotoxic (**−**)	**100%**	Yes (Value =0.3, limit=0.26)
	Not applicable		
Palmoxirate	Non-cardiotoxic (**−**)	**60**	No (Value =0.21, limit=0.26)
	Not applicable		
Panalrestat	Non-cardiotoxic (**−**)	**60**	Yes (Value =0.27, limit=0.26)
	Not applicable		
Phenformin	**Potential cardiotoxic (+)**	**70**	Yes (Value =0.26, limit=0.26)
	**Strong/extreme**	**60**	
Pioglitazone	**Potential cardiotoxic (+)**	**50**	Yes (Value =0.27, limit=0.26)
	**Weak/moderate**	**60**	
Pirogliride	Non-cardiotoxic (**−**)	**50**	Yes (Value =0.24, limit=0.26)
	Not applicable		
Ranirestat	**Potential cardiotoxic (+)**	**50**	No (Value =0.24, limit=0.26)
	**Weak/moderate**	**50**	
Remogliflozin	**Potential cardiotoxic (+)**	**50**	No (Value =0.24, limit=0.26)
	**Weak/moderate**	**50**	
Repaglinide	**Potential cardiotoxic (+)**	**50**	Yes (Value =0.31, limit=0.26)
	**Weak/moderate**	**60**	
Rivoglitazone	Non-cardiotoxic (-)		Yes (Value =0.29, limit=0.26)
	Not applicable		
Rosiglitazone	**Potential cardiotoxic (+)**	**50**	Yes (Value =0.28, limit=0.26)
	**Weak/moderate**	**60**	
Salfredin	Non-cardiotoxic (**−**)	**70%**	No (Value =0.22, limit=0.26)
	Not applicable		
Saxagliptin	Non-cardiotoxic (**−**)	**80**	No (Value =0.19, limit=0.26)
	Not applicable		
Sergliflozin	**Potential cardiotoxic (+)**	**50**	Yes (Value =0.28, limit=0.26)
	**Weak/moderate**	**50**	
Sitagliptin	Non-cardiotoxic (**−**)	**90**	Yes (Value =0.32, limit=0.26)
	Not applicable		
Sorbinil	Non-cardiotoxic (**−**)	**60**	No (Value =0.25, limit=0.26)
	Not applicable		
Sotagliflozin	**Potential cardiotoxic (+)**	**70**	Yes (Value =0.29, limit=0.26)
	**Weak/moderate**	**50**	
Sulfanilamide	Non-cardiotoxic (**−**)	**80**	No (Value =0.21, limit=0.26)
	Not applicable		
Sulfisoxazole	Non-cardiotoxic (**−**)	**70**	No (Value =0.24, limit=0.26)
	Not applicable		
Teneligliptin	**Potential cardiotoxic (+)**	**50**	No (Value =0.25, limit=0.26)
	**Weak/moderate**	**50**	
Tofogliflozin	**Potential cardiotoxic (+)**	**70**	No (Value =0.22, limit=0.26)
	**Strong/extreme**	**50**	
Tolazamide	**Non-cardiotoxic (−)**	**70**	Yes (Value =0.34, limit=0.26)
	**Not applicable**		
Tolrestat	**Potential cardiotoxic (+)**	**50**	Yes (Value =0.28, limit=0.26)
	**Weak/moderate**	**70**	
Tolbutamide	**Non-cardiotoxic (−)**	**70**	Yes (Value =0.29, limit=0.26)
	**Not applicable**		
Trelagliptin	**Potential cardiotoxic (+)**	**50**	Yes (Value =0.36, limit=0.26)
	**Weak/moderate**	**60**	
Vildagliptin	**Non-cardiotoxic (−)**	**70**	No (Value =0.24, limit=0.26)
	**Not applicable**		
Troglitazone	**Potential cardiotoxic (+)**	**60**	No (Value =0.24, limit=0.26)
	**Weak/moderate**	**50**	
Zenarestat	Non-cardiotoxic (**−**)		No (Value =0.24, limit=0.26)
	Not applicable		
Voglibose	**Non-cardiotoxic (−)**	**60**	No (Value =0.17, limit=0.26)
	**Not applicable**		
Zopolrestat	**Non-cardiotoxic (−)**	**50**	No (Value =0.28, limit=0.26)
	**Not applicable**		

### Computational studies

#### hERG inhibitory screening

Pred-hERG is a web-accessible computational tool to predict putative blockers of hERG and was used to predict the blockers and non-blockers of the hERG channels. The app is publicly available at http://labmol.farmacia.ufg.br/predherg/ [13, 23].

#### Molecular docking studies

### Protein preparation

The three-dimensional (3D) structures of the wild type of hERG channel (PDB ID: 5VA1) and mutated type of hERG channel (PDB ID: 5VA3) were downloaded from Protein Data Bank (PDB). Biovia Discovery Studio (DS) has been used to remove the co-crystallized ligands and save the protein structures and the co-crystallized ligands in the pdb format. Then, the protein structures were imported to the AutoDock where AutoDock Tools (ADT) 1.5.6 were used to remove the water molecules and add the hydrogen atoms and Gasteiger charges. Finally, the protein structures were saved in .pdbqt format.

### Ligand preparation

The 2D structures of the compounds were prepared using ChemSketch and then converted to their respective 3D structures using the Biovia DS. Then, the 3D structures of the ligands were imported into AutoDock where ADT was used to prepare the ligands for docking by adding charges, set the rotatable bonds, and allow all the torsions to rotate for the ligands. Finally, all the ligands were saved in pdbqt format.

### Molecular docking

After preparing the protein structures and the ligands for docking, the grid box and the input parameter file were prepared. The ADT was used to determine the size and coordinate of the Grid Box that covers the important amino acids in the binding pocket of the protein. Then, the size and coordinate of the Grid Box (30 × 30 × 30 Å and exhaustiveness 8) were saved in the input parameter file. Molecular docking was performed using AutoDock Vina 1.1.2. The prepared ligands were docked into the binding sites of the prepared proteins. All the docking parameters have remained as default settings (grid box 30 × 30 × 30 Angstrom and exhaustiveness 8). The binding energies were recorded and compared; the lower values of binding energy indicated stronger binding affinity with the active site of the respective protein targets. The binding poses of the ligands and the mode of interactions of the protein-ligand complex were studied using LigandScout 4.4.

## Results

### Data set

A dataset comprising of FDA-approved drugs which are used in the treatment of diabetes mellitus has been constituted (Table 1). Chemical structures, mechanism of action, pharmacokinetic profile, and side effects have been well studied and reported in the literature. The search strategy was developed for the selected database (PubMed/MEDLINE/EMBASE/Google Scholar), and all search results were combined into a file. Duplicate citations were removed, and a list of potentially relevant papers was created. The information relevant to chemical structure, physicochemical properties, antidiabetic properties, and FDA-approval status was extracted from the collected references. It is organized in alphabetical order for convenience (Table 1).

### Computational studies

#### hERG inhibitory screening

PredhERG that allows users to predict blockers and non-blockers of the hERG channels, and important drug anti-target associated with lethal cardiac arrhythmia. This app has a fast and intuitive interface. It has implemented binary (blocker vs. non-blocker) and multi-classification models, which are able to distinguish weak/moderate and strong/extreme blockers. It also implemented the probability maps of atomic contribution as predicted by the models, allowing users to interpret the results and propose structural modifications for the predicted compound. The current version of the app (v. 4.0) was developed using ChEMBL54 version 23, containing 8134 compounds with hERG blockage data after curation. This app is publicly available on the website.

#### Molecular docking studies

The comparison of the binding energies of the compounds to the selected receptor targets (PDB ID: 5VA1 and 5VA3) is shown in Table 2. This comparison allowed the identification of compounds that have high affinities to the two receptors. These compounds are Bromocriptine, Balaglitazone, and Darglitazone with binding energies ≤ 7.0 Kcal/mol. However, only Bromocriptine and Darglitazone showed consistent results with both receptors. Thus, the interactions of these two compounds with the active site residues of each receptor will be studied thoroughly.

**Table 2:** The binding energies of the inhibitors with the two receptors (PDB IDs: 5VA1 and 5VA3)

Compound name	Chemical structure	Binding energy (Kcal/mol)	
		PDB ID: 5VA1	PDB ID: 5VA3
Acarbose		**−**6.1	**−**6.2
Bromocriptine		**−**7.3	**−**7.3
Balaglitazone		**−**7.0	**−**7.2
Darglitazone		**−**7.2	**−**7.2
Dapagliflozin		**−**6.1	**−**6.6
Empagliflozin		**−**6.3	**−**6.5
Englitazone		**−**6.8	**−**7.2
Ipragliflozin		**−**6.79	**−**7.2
Lobeglitazone		**−**5.9	**−**6.3
Luseogliflozin		**−**6.0	**−**5.9
Meglitinide		**−**6.4	**−**6.0
Mifepristone		**−**6.8	**−**6.5
Phenformin		**−**5.5	**−**5.7
Pioglitazone		**−**6.1	**−**6.0
Ranirestat		**−**6.5	**−**6.5
Repaglinide		**−**6.5	**−**6.1
Remogliflozin		**−**5.7	**−**5.8
Rosiglitazone		**−**5.9	**−**5.4
Sergliflozin		**−**5.9	**−**5.8
Sotagliflozin		**−**6.3	**−**6.7
Teneligliptin		**−**6.2	**−**6.2
Tofogliflozin		**−**6.7	**−**7.0
Tolrestat		**−**6.9	**−**5.7
Trelagliptin		**−**6.0	**−**6.3
Troglitazone		**−**6.7	**−**7.5

**Fig. 2:**
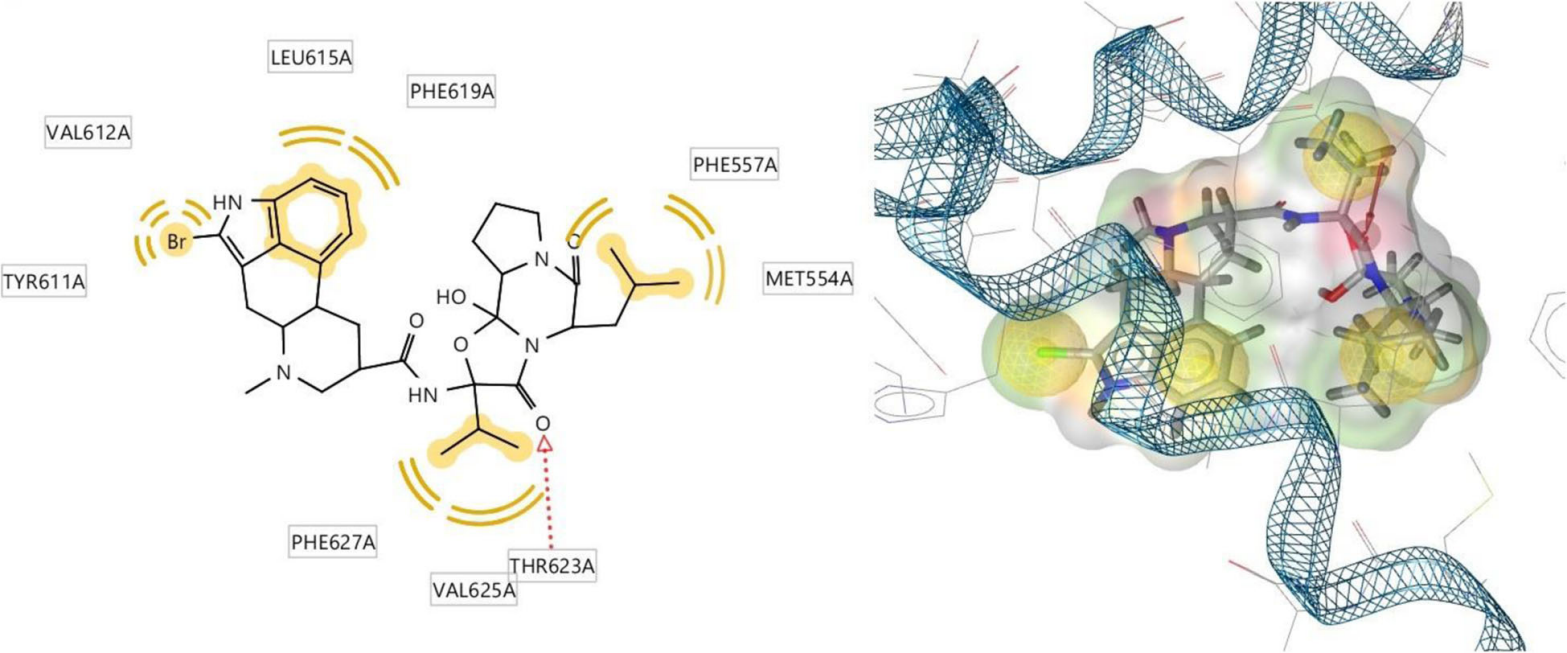
The binding mode of Bromocriptine within the active site of 5VA1. The hydrogen bond is presented in red dotted line.

**Fig. 3:**
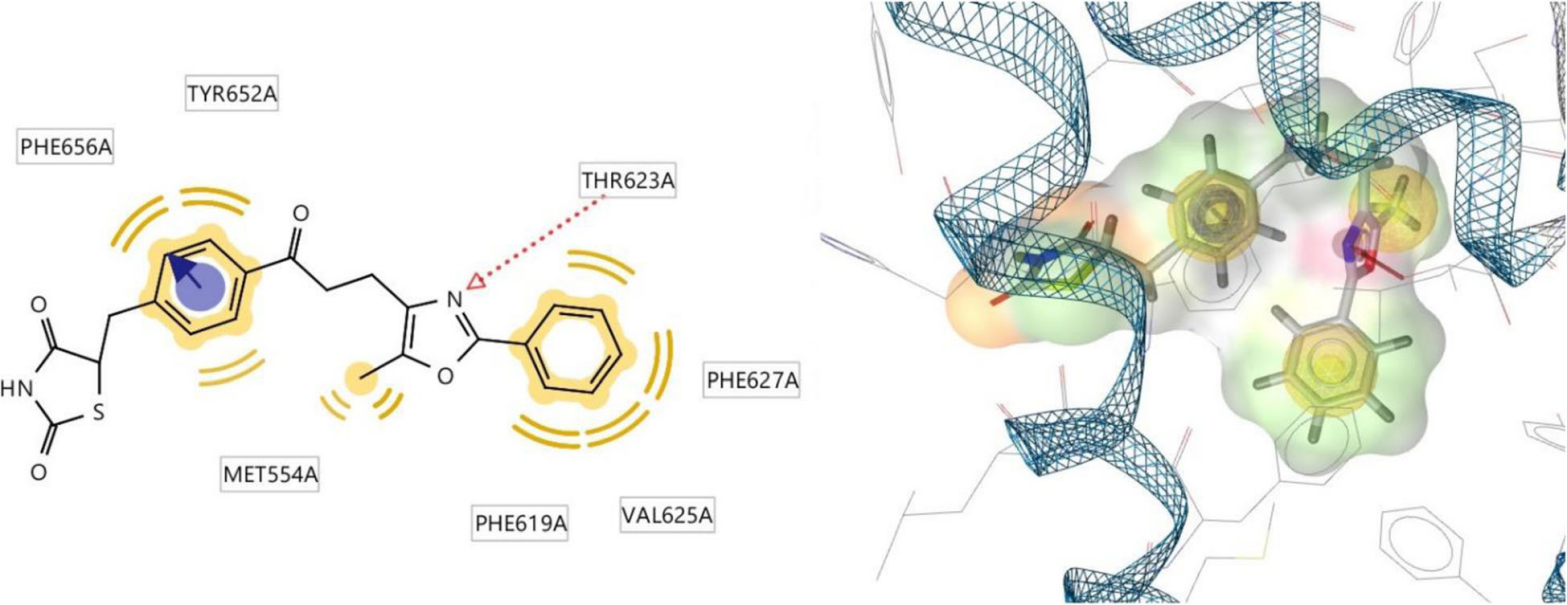
The binding mode of Darglitazone within the active site of 5VA1. The hydrogen bond is presented in red-dotted line and aromatic interaction in blue.

**Fig. 4:**
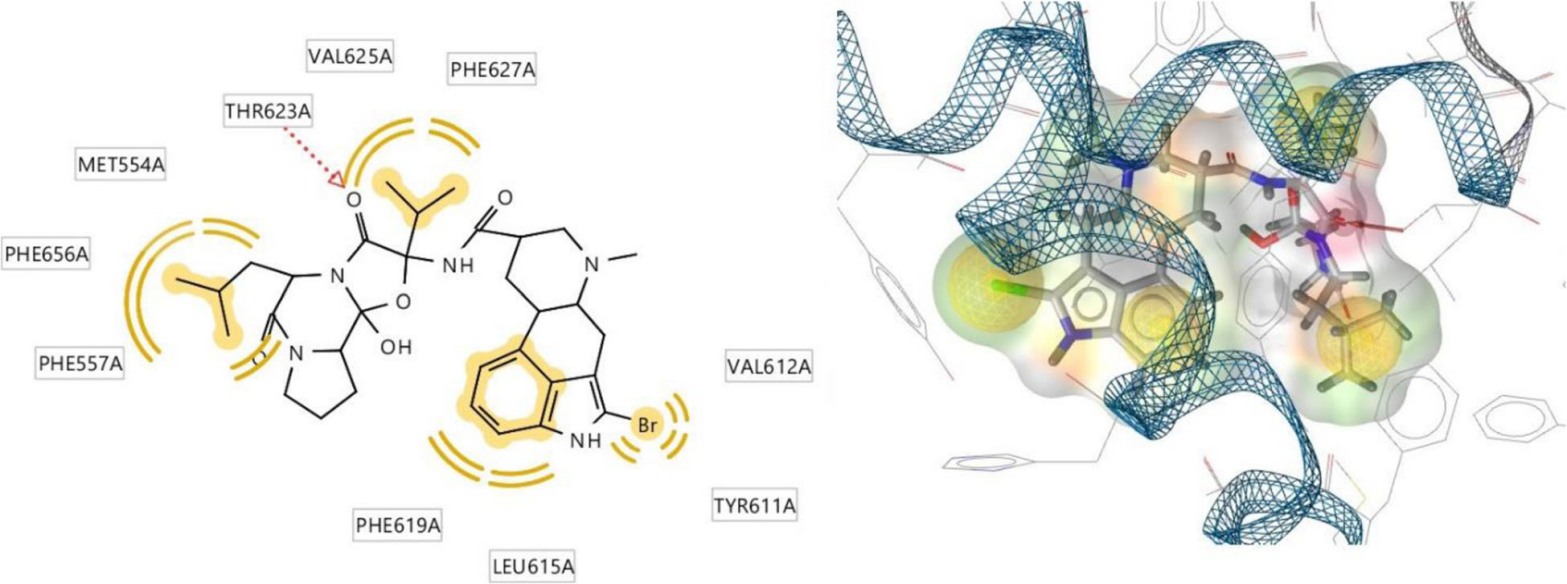
The binding mode of Bromocriptine within the active site of 5VA3. The hydrogen bond is presented in red-dotted line.

**Fig. 5:**
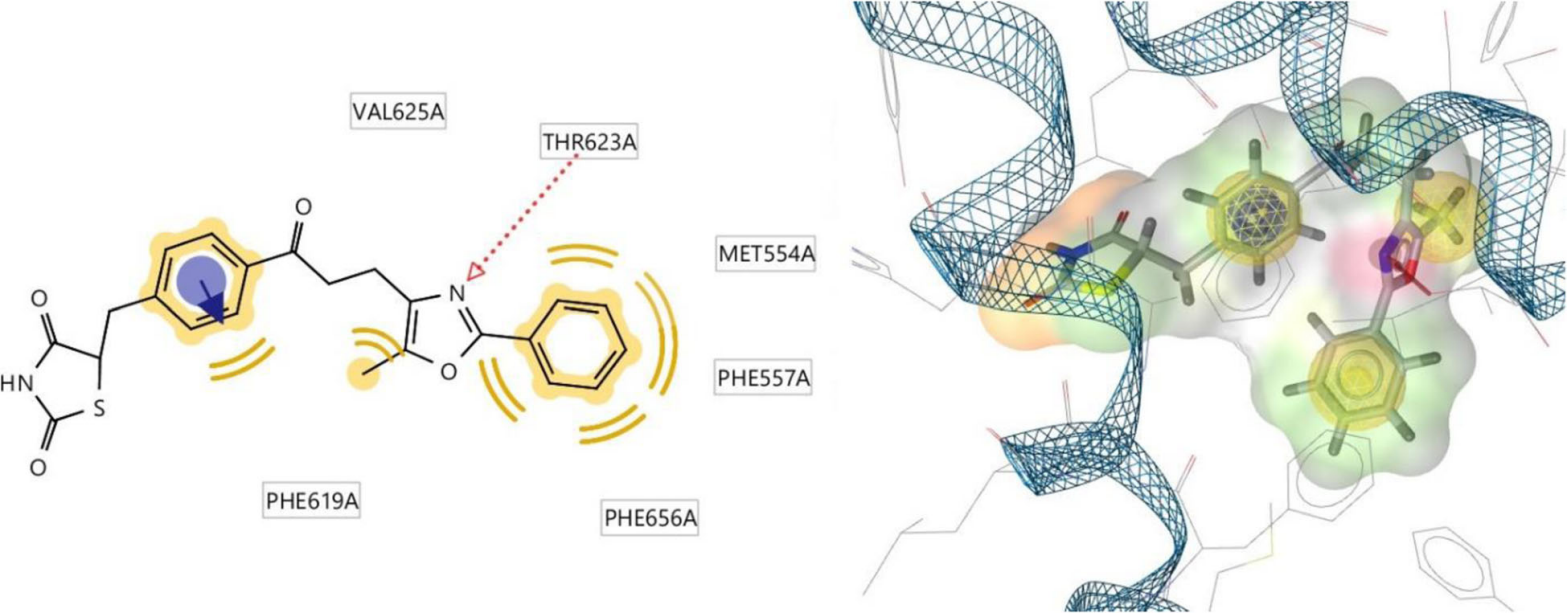
The binding mode of Darglitazone within the active site of 5VA3. The hydrogen bond is presented in red-dotted line and aromatic interaction in blue

**Fig. 6:**
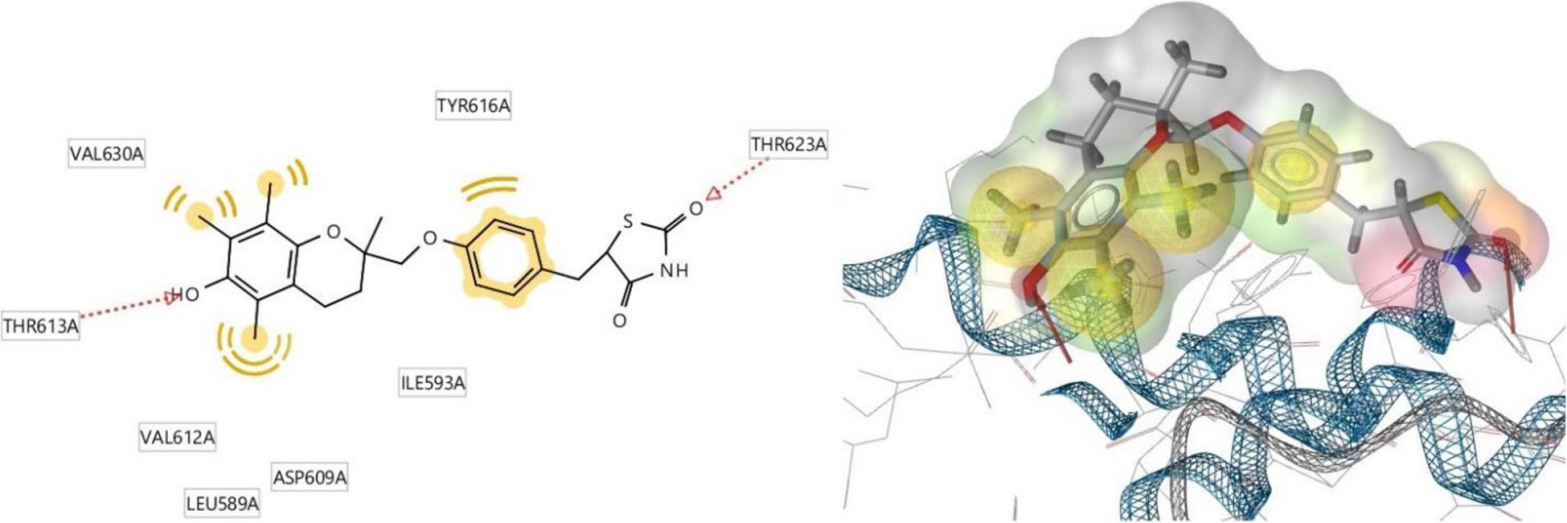
The binding mode of Troglitazone within the active site of 5VA3. The hydrogen bonds are presented in red-dotted lines

The docking study of the selected set of compounds displayed the best binding pose of the respective compounds within the binding sites of 5VA1 and 5VA3, elucidated the interactions involved in the binding of the compounds with the receptors, and showed the effect of the mutation on the affinity of the compounds to the target receptor (Table 2). The binding interactions for high-affinity compounds namely, Bromocriptine, Balaglitazone, Darglitazone, and Troglitazone are shown in Figs. 2, 3, 4, 5, and 6.

## Discussion

Over the past few decades, research efforts have successfully contributed to identify potential anti-diabetic agents having FDA approval for clinical use. A dataset comprising clinically approved anti-diabetic agents was constructed, and the details of chemical structures, mechanism of action, pharmacokinetic profile, and side effects have been collected.

In human cancer cells, the hERG is often overexpressed on their plasma membrane and regulates proliferation, survival, migration/invasiveness, and neoangiogenesis. Thus, selective inhibitors of hERG can be used as anticancer therapeutics [24, 25]. A similar role of hERG on glioblastoma has been reported by Pointer and colleagues and suggests the application of similar strategies to control other types of cancers. The publicly available PredhERG application was used to predict blockers and non-blockers of the hERG channels from the constructed dataset of anti-diabetic agents. The binary and multi-classification models could significantly classify blockers and non-blockers of hERG which is an important drug anti-target associated with lethal cardiac arrhythmia.

The molecular docking study was performed using the reported molecular structure of hERG_T_ and hERG_TS_ (3.8 Å) (PDB IDs: 5VA2 and 5VA1, respectively) [Wanfg et al., 2017]. The study reports depolarized conformation (open and voltage sensors) of the channel and presence of a unique central cavity which contributes to hERG block by many drugs. The pockets are not present in other K^+^ channels, and the small volume favors the stable binding of cationic drugs and amplifies the electrostatic potential. The asymmetric binding of drugs causes partial occupancy of the functional groups in the hydrophobic pockets.

The docking study of the selected set of compounds displayed the best binding pose of the respective compounds within the active sites of 5VA1 and 5VA3, elucidated the interactions involved in the binding of the compounds with the receptors and showed the effect of the mutation on the affinity of the compounds to the target receptor (Table 2).

The putative drug-binding site of 5VA1 includes the following residues: THR623, SER624, and VAL625 on the pore helix and residues GLY648, TYR652, and PHE656, located on segment S. Bromocriptine and Darglitazone occupied the active site of the receptor and interacted with the active site’s residues through hydrogen bond and hydrophobic interactions. For Bromocriptine, the hydrogen bond formed between the oxygen atom of the ligand and the residue THR623 (Fig. 2) while the amino group of Darglitazone was involved in a hydrogen bond with THR623 (Fig 3). Both ligands formed hydrophobic interactions with the residues MET554, PHE619, and VAL625. Additionally, the phenyl group of Darglitazone displayed aromatic interaction with the aromatic amino acid PHE656.

The second selected receptor 5VA3 is the mutated type of hERG channel in which the residue SER631 was replaced with ALA631. The binding mode of Bromocriptine and Balaglitazone within the active site of 5VA3 was like their binding mode within the active site of 5VA1. Both the compounds showed interactions with 5VA3 similar to that with 5VA1. The two compounds formed hydrogen bonds with THR623 and formed hydrophobic interactions with MET554, PHE619, and VAL625 in addition to PHE557 and PHE656 (Fig. 4). The phenyl group of Darglitazone displayed aromatic interaction with the aromatic amino acid PHE619 (Fig. 5).

Although Bromocriptine and Darglitazone exhibited low binding energies to 5VA3, these two compounds did not show the highest affinity for 5VA3. As can be seen in Table 2, Troglitazone showed the lowest binding energy to 5VA3 (−7.5 Kcal/mol) amongst all the 26 compounds. Therefore, this compound has the highest affinity for the target receptor. Troglitazone is involved in two hydrogen bonds with the residues THR613 and THR623. These hydrogen bonds formed between the two oxygen atoms of the ligand and THR613 and THR623, respectively (Fig. 6). The oxygen atoms acted as hydrogen bond acceptors. Moreover, the ligand was also involved in hydrophobic interactions with LEU589, ILE593, ASP609, VAL612, TYR616, and VAL630.

The docking study has shown the main amino acids that are essential for the binding of the ligand to the target receptors. These amino acids are THR623 and VAL625. Besides, it has been shown that replacing SER631 with LAL631 did not affect the binding affinity of most of the compounds except Troglitazone which showed a higher affinity to the mutated protein than the wild type.

When the derived results were compared with the previously reported data, we found anti-cancer potential of some of the selected compounds. Cytotoxic potential of the selected four hERG blockers namely, Bromocriptine, Balaglitazone, Darglitazone, and Troglitazone have been reported through in vitro/in vivo experimental methods in various cancer types. Bromocriptine, a sympatholytic, D2-dopamine agonist used for the treatment of type 2 diabetes [26] has been investigated for the treatment of metastatic breast cancer, prostate cancer-related hyperprolactinemia [27]. Bromocriptine has a cytotoxic profile towards drug-sensitive CCRF-CEM, multidrug-resistant (MDR) CEM/ADR5000 leukemic cells, and MDR ABCB5-transfected HEK293 cell lines. The proposed mechanism for cytotoxicity includes binding to NF-_K_B proteins [28]. The next antidiabetic agent Balaglitazone, a second-generation peroxisome proliferator-activated receptor-gamma (PPAR-γ) agonist, could reverse P-glycoprotein-mediated MDR by upregulating phosphatase and tensin homolog deleted on chromosome 10 (PTEN) in leukemia cells [29]. The PPAR-γ agonists (Troglitazone) have been investigated as a combination with other drugs such as lovastatin in human anaplastic thyroid cancer cell line and in a mouse xenograft model [30]. Troglitazone in combination with aspirin has shown cell cycle arrest and apoptosis in human lung cancer cells [31]. In a recent study, Troglitazone has significantly suppressed the growth of human oral SCC cells but failed to induce apoptosis [32]. In pancreatic cancer, troglitazone has shown cytotoxicity via the JNK pathway and mitochondria-mediated apoptosis which was independent of PPAR-γ. The study reports the absence of marked adverse effects during the in vivo antitumor investigation [33].

## Conclusion

The selected blockers of hERG K+ channel were docked into the near-atomic resolution cryo-EM structures of the hERG wild type (WT) (PDB ID: 5VA1, 3.7 Å resolution) and S631A mutant channels (PDB ID: 5VA3, 4.0 Å resolution). The computational approach based on the prediction of hERG blockers/non-blockers and molecular docking properties could help to investigate the proposed mechanism for anti-cancer activity of the selected non-cancer agents.

The study for investigation of non-cancer hERG blockers as potential anti-cancer therapeutics using in silico methods was successfully completed. The proposed mechanism for cancer inhibitory properties via hERG blocking for compounds Bromocriptine, Balaglitazone, Darglitazone, and Troglitazone should be further explored using other experimental methodologies. The non-cancer to anti-cancer approach may direct to provide a therapeutic solution to late-stage cancer and benefit the cancer patients.

## Data Availability

Not applicable

## *Abbreviations*

ADT: AutoDock tools
DPP IV: Dipeptidyl peptidase IV
GLP-1: Glucagon-like peptide-1
hERG: Human ether-a-go-go-related gene
JNK: C-Jun N-terminal kinase
LQTS: Long QT syndrome
MDR: Multidrug-resistant
PDB: Protein data bank
PPAR-γ: Peroxisome proliferator-activated receptor gamma
PTEN: Phosphatase and tensin homolog deleted on chromosome 10
SCC: Squamous cell carcinoma
US FDA: United States food and drug administration
